# Evaluating the effectiveness and limitations of online health information tools in assessing the quality of medication-related content

**DOI:** 10.3389/fpubh.2025.1460202

**Published:** 2025-02-24

**Authors:** Adel S. Alhlayl, Haitham A. Alzghaibi

**Affiliations:** ^1^Department of Continuous Education at the Executive Directorate of Training and Research Affairs, Hail Health Cluster, Hail, Saudi Arabia; ^2^Department of Health Informatics, College of Applied Medical Sciences, Qassim University, Buraydah, Saudi Arabia

**Keywords:** amitriptyline, online health information, reliability testing, medication information, web-based assessment tools, keystone criteria

## Abstract

**Introduction:**

The internet has become a primary source of information on medicines, yet the quality of this information is inconsistent. Despite the proliferation of web-based resources, limited research has specifically examined the reliability of online information on medicines. The variability in quality can be attributed to the recent shift toward digital information-seeking and the absence of specialized tools designed to assess the quality of medication-related information online.

**Aim:**

To evaluate the quality of information about Amitriptyline available on websites using three well-established, generic evaluation tools—HONcode, DISCERN, and JAMA—compared against the domain-specific Keystone action plan criteria.

**Methods:**

Adopting an evaluative research design, this study utilized the aforementioned four assessment tools to collect and analyze data. Four independent assessors were recruited to evaluate the quality of the information on selected websites.

**Results:**

A Google search using “Amitriptyline” identified 30 websites for the study sample. Reliability analysis using Cronbach’s alpha, the Inter-Class Correlation coefficient, and Kendall’s rank-order correlation coefficient revealed that the Keystone criteria demonstrated the highest internal consistency and reliability compared to DISCERN, HONcode, and JAMA.

**Conclusion:**

This study indicates that the Keystone action plan criteria may serve as a reliable, domain-specific tool for evaluating medicines information on websites. The results underscore the importance of specialized assessment criteria for online medication information, as they provide more consistent and accurate evaluations compared to generic tools.

## Introduction

In last decade, the internet has become a primary source of health information, providing a vast array of materials on several treatments. However, the quality and accuracy of this information frequently exhibit inconsistency, prompting questions regarding the credibility of online sources (1–3). The internet has revolutionized access to medical knowledge; nonetheless, the surge of unregulated content presents concerns, particularly for intricate subjects such as medication usage. Assessing the quality of internet health information has become essential to guarantee that customers receive trustworthy and safe advice (1–6).

Notwithstanding the plethora of internet resources, scant research has concentrated specifically on the quality of web-based drug information. A potential reason for this neglect could be the increasing prominence of internet-based health information-seeking activities, which has become more widespread in recent years. Although tools for evaluating online health information have existed for some time, including those available as early as 2002, the lack of specialized techniques specifically for assessing online medication-related information has hindered more rigorous and consistent evaluations (7). The gap is particularly alarming as erroneous or inadequate drug information can lead to significant health consequences, particularly when people depend on online sources without seeking guidance from healthcare professionals (7, 8). Initiatives to evaluate and enhance the quality of online consumer health information have resulted in the creation of numerous assessment tools. DISCERN, created in 1996–1997, was among the first tools intended to assist consumers in differentiating between high-and low-quality health information online. This technique, among others, established a basis for evaluating the dependability and the credibility of online health information. Nonetheless, the majority of these instruments are comprehensive, designed to assess general health information without a particular emphasis on medicine (9–13).

After the introduction of DISCERN Jadad and Gagliardi (14), Gagliardi and Jadad (15) discovered 47 distinct methods for assessing the quality of online health information, a figure that increased to 98 by 2002. After the introduction of DISCERN (16). Zhang et al. (17) highlighted that, despite the plethora of evaluation tools accessible, a mere minority have passed stringent reliability testing, and the majority are not intended to analyze medication-specific information. The absence of targeted assessment tools is a considerable constraint, as broad health evaluation criteria may fail to encompass the specific elements of drug information, including dosage recommendations, side effects, and interactions (17). The majority of the developed assessment tools were generic, intended to evaluate diverse categories of health information, including diseases, fitness, and nutrition, rather than being specifically customized for the evaluation of medicinal information on websites. Although these tools are extensively utilized, their efficacy in assessing medicines-related material remains ambiguous, and there is scant data regarding their success rates in this specific area.

In accordance with Public Law 104–180, which mandated the U.S. Department of Health and Human Services to improve public access to high-quality health information, the Keystone Center established standards for evaluating consumer pharmaceutical information. This action plan delineated fundamental principles, indicating that drug information must be scientifically correct, objective, specific, thorough, comprehensible, current, and practical (18). These guidelines were established to provide a dependable framework for assessing medication-related material. A scoring system was implemented to enable assessors to evaluate the degree to which each condition is satisfied. Nonetheless, despite its specific design, the Keystone criteria have been utilized in only a restricted number of studies and have not experienced thorough reliability assessment (18).

The Keystone action plan was developed with criteria for evaluating useful consumer medicines information, composed of 12 items. Kim et al. (18) assigned a gradation scale to these criteria. The scale determines whether each criterion is fully met (2 points), partially met (1 point), or not met (0 points), with 2 points deducted if the information is inaccurate. The authors applied this scale to evaluate three different websites—Medline Plus, Yahoo Health, and WebMD—resulting in varying assessment scores.

In this context, reliability denotes the consistency and dependability of an information source (19). Credibility is a fundamental element of information quality, as consumers frequently associate it with quality (20–24). Internet users who often seek health information online are more attuned to the perceived credibility of sites. Research has continuously demonstrated a robust correlation between source credibility and user assessments of quality (21). The significance of this interaction is heightened in the realm of pharmaceutical knowledge, since individuals may depend on internet resources to make treatment and drug decisions without seeking guidance from a healthcare professional.

Current research indicates that internet health information frequently suffers from deficiencies in both quality and credibility (7, 25, 26). Akbari and Som (27) indicated that sampled webpages concerning diverse health subjects had typically inadequate quality and believability. Bates et al. (21) discovered that although consumers generally accept health information from websites linked to trustworthy organizations, the quality of this information might vary significantly. The pervasive presence of substandard drug information online highlights the necessity for dependable, specialized evaluation instruments that assist consumers in discerning credible sites. Kwon et al. (28) surveyed online health information seeking and showed that most participants placed a large amount of trust in websites established by health organizations.

There is no indication of the quality of medical information online, which presumably varies from website to website. Patients are not likely to describe the Internet materials to their doctors or bring these materials into clinic visits typically (29, 30). Doctors rarely advise their patients to search health-related material on the Internet (30). The Royal Pharmaceutical Society of Great Britain (RPSGB) has reported that over 2 million people in the United Kingdom purchase medicines via the Internet on a regular basis (31). The first case of poisoning by a product purchased through the Internet was reported in the New England Journal of Medicine (32). Still, there might be some unreported cases of harm or increase in the cases of hazards in future (30, 33, 34). Raine et al. (35) discovered 96 online pharmacy websites that sold medication to the United Kingdom population, including prescription only drugs, with a shortage of online information such as directions of use or side effects.

This study seeks to rectify these deficiencies by assessing the quality of information regarding Amitriptyline, a commonly prescribed antidepressant. Amitriptyline is frequently used to treat conditions such as neuropathic pain in multiple sclerosis (MS) and depression. It is one of the many medications for which patients actively seek information online (36). Amitriptyline is a critical subject of research in the context of online health information, where patients and caregivers often seek reliable guidance regarding its use, side effects, and interactions with other medications. From where you get this statement (37). The selection of Amitriptyline for this study is justified by its widespread use and the substantial amount of online content available about it (36). Its therapeutic applications for various conditions, including gastrointestinal disorders, fibromyalgia, and headaches, highlight the need for accurate and reliable information to support both patients and healthcare providers (37). Furthermore, the extensive availability of online content underscores the importance of assessing the quality of the information provided (36).

Several factors make Amitriptyline an ideal subject for this study. First, Amitriptyline is one of the most commonly prescribed antidepressants for patients with multiple sclerosis (MS), as well as for individuals with depression. It is often used initially for managing neuropathic pain in MS patients and then as an antidepressant to address the high prevalence of depression in this population (38, 39). Research shows that Amitriptyline was cited in 17.5% of suicidality-related coroners’ inquests in England and Wales between 2003 and 2020, highlighting its significant role in both mental health and medication safety discussions (40). Second, Amitriptyline is increasingly being used in the treatment of other conditions such as gastrointestinal ailments, fibromyalgia, and headaches (38). In fact, Amitriptyline accounted for 157,516,372 daily doses prescribed in England and Wales during 2017–2018, demonstrating its widespread use in clinical practice (41).

Despite the broad availability of Amitriptyline-related content online, much of this information is not consistently accurate or reliable. Therefore, it is essential to evaluate the tools that individuals use to assess the quality of online medication-related information. By doing so, this study aims to highlight the challenges of ensuring the accuracy and reliability of online content and provide a more robust understanding of how such tools can be used to support informed healthcare decisions.

## Aim and objectives

### Study aims

The aim of this study is to evaluate the quality of information about Amitriptyline available on websites using three of the existing well-known generic evaluation tools HONcode, DISCERN and JAMA against the web scoring scale for the Keystone action plan criteria used by (18). The results from these tools will then be compared so that the reliability of this web scoring scale can be estimated.

### Objectives

1. To evaluate the quality of information about Amitriptyline available on websites.
2. To measure the reliability of the Keystone action plan criteria web scoring scale against the results obtained using the HONcode, DISCERN, and JAMA generic tools.

## Methods

### Design

This study follows an evaluative research design, using four assessment tools mentioned above to make data collection. The assessment results were obtained by using four recruited independent human assessors.

### Population and sample

A sampling strategy was applied to select websites for evaluation that contain information about Amitriptyline. A total of 30 web pages were used. A number of 30 observations collected as a sample size would be appropriate for analysis and for detecting statistical significance based on previous studies (42, 43). The websites have been collected using the Google search engine. This is because it was reported that Internet users operate Google as their most commonly used search-engine (44–46). In the Europe, about 85% of people who use the Internet launch Google to seek information (47). Google usually produces overlapping search results output with other search engines (6, 47, 48). Agreeing with the above citations, the survey outcomes obtained in Alhlayl (39) confirmed that the participants mainly use search engines such as Google to find information related to MS and medicines they consume.

On the other hand, the inclusion of the websites was based primarily on their popularity, according to their ranking in the list of search results. Then the following inclusion and exclusion criteria were applied:

Websites were included if they:

1. Were written in English.
2. Were aimed at the general public (i.e., not purposely designed for health professionals).
3. Provided substantial information about Amitriptyline.

However, websites were excluded if they:

1. Were inaccessible (i.e., could not be accessed due to a link fault or a password protection).
2. Repeated other websites already included in the sample.
3. Referred to only articles, books, blogs, or slideshows.

From the total hits yielded by entering ‘Amitriptyline’ as a keyword in the Google search engine, 42 websites were initially identified for the study sample. Of these, six websites were excluded because they did not contain substantial information, four were excluded as duplicates, and two were excluded due to inaccessibility. Therefore, 30 websites were selected as the sample for this study.

### Criteria used to determine that the information on the included websites

Websites were evaluated according to predefined criteria for “substantial” content concerning Amitriptyline to guarantee the inclusion of only pertinent and thorough information in the analysis. Initially, each website was required to encompass essential prescription information, including the uses of Amitriptyline, dose recommendations, adverse effects, interactions, and contraindications. Sites that failed to furnish all these required components were omitted. Additionally, the level and complexity of the content were evaluated; only websites offering thorough explanations of Amitriptyline’s mechanism, therapeutic effects, and supporting evidence were deemed substantial. This study concentrated on consumer health information, necessitating the content to be articulated in a simple and accessible manner for the general public, eschewing technical language intended for healthcare professionals. These criteria guaranteed that the selected websites offered a dependable and thorough resource for assessing the quality of Amitriptyline-related material accessible to consumers online.

### Data collection instruments

Zhang et al. (17) classified the types of tools operated to evaluate medical information on the web into generic and domain-specific instruments. According to Zhang et al. (17), Fahy et al. (49), and Al-Jefri et al. (50) HON principles, DISCERN instrument and the Journal of the American Medical Association (JAMA) benchmarks are the most used generic instruments. Therefore, the data was collected using these generic instruments in addition to the Keystone action plan criteria as the domain specific instrument. In this way a comparative analysis can be conducted between the results of all these tools.

The DISCERN instrument is a commonly applied scale for quality evaluation of health information online (9). The scale is comprised of 16 questions, each of them worth 1 to 5 Likert scale points. Section 1 evaluates reliability of the investigated webpage by 8 questions while section 2 assesses the quality of treatment information by 7 questions. The last section is composed of only one question that provides an overall judgment of the webpage being examined based on the mistakes found in previous questions (9). The DISCERN instrument was tested for reliability and validity to evaluate the quality of written information about health (9). The inter-rater agreement was calculated using Cohen’s kappa (*κ*) with participators taking part in each development phase with a κ result of 0.56 (9, 51). DISCERN was created by interacting processes including the NHS Center for Reviews and Disseminations, medical press members, nurses, plain English advocates, doctors, lay health information publishing groups, the community health council, specialists on health information consumer, and delegates on self-help groups (52).

DISCERN was developed to assess printed documents but it is appropriate for web-based information assessment given that information available on the internet is in written format and read sometimes in print layouts (51, 53). DISCERN was operated to create and gage health information in web based and paper-based treatment leaflets as it revolves around treatment choices (51, 54, 55). The NHS Executive Anglia and Oxford Research and Development Program was funding a new plan to make DISCERN available and workable to all online information seekers about treatment choices (54, 55). The DISCERN instrument can estimate the credibility and reliability features of a webpage like benefits or risks of treatment and non-treatment options, publishing dates, and apparent sources of information (55). The DISCERN total score varies from 0 to 80 points, with higher scores representing a higher quality site.

The HON foundation in Geneva developed a code of conduct (HONcode) of eight principles to enhance the reliability of health information published online (56–58). These principles were reformed with numerical scales by many researchers such as Boyer (58), Ahmed (59), and Rothrock et al. (60). Ahmed’s adaptation of these principles into a questionnaire with a 1 to 5 Likert scale was chosen. The questionnaire comprised 15 queries that guarantee the covering of the HONcode 8 principles.

The JAMA benchmark was reported by Zhang et al. (17) has 4 criteria that allow readers to estimate the credibility, reasonability, and usefulness of health information found on the web. The criteria score ranges from 0 to 4 by awarding a point for clear presentation of the following core standards authorship, attribution, disclosure, and currency.

### Procedure

The sampled websites were saved as HTML files and stored in a OneNote portfolio. The portfolio was composed of four parts and each part has its own password to make sure that participants are blinded to each other’s score. Four human independent assessors as “evaluators” with different levels of educational attainment were recruited to participate in this study to evaluate the sampled websites one by one. The participants were two female medical school students (undergraduate and postgraduate), one employed man who has completed an A level education and one unemployed man who completed secondary education between 14 and 16 years of age. The participants were initially trained and calibrated regularly for 2 weeks prior starting the assessment mission. It is important to note that no familial, professional, or communal affiliations exist between the authors and the raters in this study. The evaluators were chosen for their pertinent expertise, and their evaluations were performed independently to ensure objectivity and reduce potential biases.

Raters were selected based on their availability during the research process and the ethical approval obtained from Swansea University. The participants, consisting of laypersons or caregivers, did not possess specialized knowledge in Amitriptyline or health information evaluation. Participants were available at Swansea University and consented to engage in training courses that lasted approximately 2 weeks. The training sought to acquaint participants with evaluation tools and criteria for assessing the quality of online medication-related content. This approach was chosen to enable objective evaluations by raters concerning the tools and their effectiveness in assessing health information, while minimizing potential biases arising from prior relationships or specialized knowledge in the field.

In 2020, the sampled websites were distributed to each assessor in an independent and confidential manner for evaluation. Each assessor received an email from the research primary supervisor containing a link to the portfolio with the password and the rating instrument instructions for DISCERN, HONcode principals, JAMA and the Keystone criteria. Each assessor was asked to store the results in the specific part of the portfolio allotted to her/him and send a notifying email to one of the authors when the assessment was completed.

### Data and statistical tests

Reliability of the Keystone action plan criteria web scoring scale was estimated by measuring the inter-rater reliability. The four assessors performed evaluation of the 30 sampled websites and inter-rater testing statistics were calculated with significance levels where appropriate reflecting the degree of agreement and disagreement between the assessors. For this purpose, Cronbach’s alpha, the Inter-class Correlation (ICC), and Kendall’s rank-order correlation coefficient tests were performed using SPSS V25.

## Results

### Demographic and background information

The sampled websites were categorized and grouped by their developers’ names into **commercial** websites (*n* = 17) including pharmaceutical companies providing information about medicines, **not for profit** organizations (*n* = 5) including national societies, private and personal developed websites, **health organizations** (*n* = 4) including health bodies and national institutes**, charities** (*n* = 2) and **academic** websites (*n* = 2) including universities and research centers. Each assessor filled out assessment worksheets containing names, URLs, and scores of the sampled websites and saved to the OneNote portfolio. The results then were tabulated for analysis.

### Psychometric testing of the reliability of the evaluation tools

This section aims to assess the internal reliability and inter-rater reliability of the four main tools: Keystone (22 items), DISCERN (16 items), HON (15 items) and finally JAMA (4 items). It should be noted here that the scoring systems of these tools vary (see appendices 3 and 12). The researcher aimed to examine the internal reliability of each as well as the inter-rater reliability using 4-moderators. It should also be noted at this early stage that the internal reliability is sensitive to the number of participants (moderators) hence outcomes must be treated with caution. These tools were used to examine 30-websites all of which provided information relevant to Amitriptyline. Hence it was also important to test the consistency of information across the websites too (internal reliability).

### Internal reliability: Cronbach’s alpha

Cronbach’s alpha is a reliability indicator related to the average correlation of scores for different measurements of the same underlying scale (61). Cronbach’s alpha is applied as a scale of “internal consistency” reliability (62). In other words, this section answers whether or not the items within each tool are correlated or consistent with each other. To do so, an SPSS file was created for each tool (4 moderators); Columns represented each item within each of the tools (e.g., keystone had 22 items). Row represented moderators’ (*n* = 4) assessment for each of the 30 websites. This created 120 rows (30 per moderator). Therefore, the internal reliability for each tool was calculated for each of the moderator separately (30 websites).

As observed in Table 1, Keystone reliability across items was relatively good for all moderators ranging between 0.64 and 0.823 for the 30 websites. However, the table shows the reliability for the other three tools did not reach an acceptable level.

**Table 1:** Internal consistency of each of the tools per each of assessor.

Moderator	Keystone (22)	DISCERN (16)	HON (15)	JAMA (4)
1	0.823	0.459	−0.408	0.566
2	0.698	−0.144	−0.073	0.162
3	0.659	−0.290	0.052	−0.097
4	0.640	0.157	0.206	−0.571

Furthermore, Cronbach’s alpha was calculated to test the consistency between the 30 websites assessment results based on that they were obtained by the four human moderators using each of the tools separately. Participants were asked to examine 30 websites and record scores based on the four assessment tools. The internal reliability was calculated to see consistency between 30-websites in their information. This was done for four participants (for each tool) across 30 websites, resulting in four reliability tests (Table 2).

**Table 2:** Internal consistency within each of the three tools.

Tools	Cronbach’s alpha	No of items
Keystone	0.936	30
DISCERN	0.701	30
Hon	0.321	30
JAMA	0.103	30

As can be seen in Table 2, Cronbach’s alpha showed that Keystone had the highest internal consistency and reliability result of (0.936) followed by DISCERN (0.701), HON (0.321) and finally JAMA (0.103). It should be acknowledged that these reliability tests were conducted based on four evaluators’ answers and this makes Cronbach’s alpha more sensitive to inconsistencies between websites.

It is worth noting that the internal consistency of each tool is reflected in the results of Table 1, which are based on the evaluations conducted by the four moderators for each of the 30 websites.” In particular, this table displays the Cronbach’s alpha values that were determined for each tool by analyzing the individual assessments of the moderators. This information offers a glimpse into the consistency with which each moderator evaluated the websites using each tool. In contrast, Table 2 displays the overall internal consistency of each tool across all 30 websites, as determined by the collective ratings provided by the four moderators. The Cronbach’s alpha value evaluates the consistency of the ratings across the websites, indicating the reliability with which each tool measures the same construct across various websites.

## Inter-rater reliability

Inter-Class Correlation Coefficient (ICC) is commonly used to calculate the degree of conformity among different observers measuring the same quantity (63). Inter-rater reliability for each of the three tools in this study was tested using ICC. ICC is an index that reflects the reliability of the ratings provided by assessors, with higher scores indicating greater inter-rater reliability. However, the interpretation of the ICC score is not solely based on the value itself; the reliability is also assessed based on established thresholds for the score. Typically, an ICC value below 0.50 indicates poor reliability, values between 0.50 and 0.75 suggest moderate reliability, values between 0.75 and 0.90 indicate good reliability, and values above 0.90 are considered excellent. In this study, ICC measures the consistency of ratings across more than two assessors.

Total scores for each tool were calculated across all websites per assessor, resulting in four columns per tool for 30 websites. Inter-class correlations (ICC) were then conducted for each tool to assess consistency among assessors. The single measures ICC represents the reliability of one assessor, while the average ICC indicates agreement across all four assessors. As shown in Table 3, the tools demonstrated high inter-rater reliability, with DISCERN (ICC = 0.996, *p* < 0.001) showing the highest consistency, followed by JAMA (ICC = 0.935, *p* < 0.001) and Keystone (ICC = 0.929, *p* < 0.001). This suggests that each tool provides clear instructions for reliable use. However, HON had low reliability (ICC = 0.149, *p* > 0.05), consistent with previous critiques of its principles (7, 53).

**Table 3:** Inter-class correlation coefficient for keystone, DISCERN and JAMA.

Tool	Intraclass Correlation (Average Measures)	95% Confidence Interval	*F* Value	Significance (Sig)
Keystone	0.929	0.876–0.963	14.06	0.000
DISCERN	0.996	0.993–0.998	256.463	0.000
HON	0.149	−1.008–0.400	0.87	0.656
JAMA	0.935	0.887–0.966	15.489	0.000

### Kendall’s rank-order correlation coefficient

Kendall’s rank-order correlation coefficient is typically used to assess the association between two ordinal variables or between an ordinal and a continuous variable, especially when the data contains tied ranks or when the sample size is small (64). In this study, Kendall’s rank-order correlation coefficient was employed to evaluate the relationship among the scores of the four instruments—Keystone, DISCERN, JAMA, and HON—because the data consisted of ordinal scores assigned by the evaluators. Kendall’s Tau is particularly advantageous in this case because it is less sensitive to tied ranks than Spearman’s rank-order correlation, making it more suitable for assessing the consistency between evaluators’ ratings of ordinal data. A cumulative score for each instrument, derived from all four evaluators, was computed for each website, producing four columns of aggregate scores corresponding to each tool across 30 observations.

Substantial positive correlations were identified between Keystone and DISCERN (τb = 0.559, *p* < 0.001), Keystone and JAMA (τb = 0.565, p < 0.001), and DISCERN and JAMA (τb = 0.782, p < 0.001), thereby affirming the validity of Keystone in alignment with other standards. No substantial relationships were detected between Keystone and HON (τb = 0.208, *p* > 0.05) or between HON and the other instruments.

Total scores were computed for each website by each assessor for the tools (Keystone, DISCERN, and JAMA), and subsequently aggregated across assessors. This yielded a singular score per tool for each of the 30 websites, facilitating a ranking from lowest to maximum quality. HON was omitted due to its minimal association with the other instruments. The rankings for DISCERN and Keystone spanned from 1 to 25, whereas JAMA varied from 1 to 4, with 1 representing the lowest score and 25 or 4 denoting the highest score.

To facilitate score comparison among instruments, Keystone and DISCERN scores were categorized into five groups (1 = 1–5, 2 = 6–10, etc.), corresponding to JAMA’s 1–4 ranking system. This alteration enhanced uniformity among the instruments. Significantly, Keystone and DISCERN exhibited identical rankings for W2, W13, and W24, with analogous patterns observed at both the high and low extremes of the scales across all instruments, in accordance with the correlation data presented in Table 4.

**Table 4:** Kendall’s Tau correlation coefficient between the three tools.

Tool	Keystone	DISCERN	JAMA	HON
Keystone	1.000	0.559**	0.565**	0.208
DISCERN	0.559**	1.000	0.782**	0.103
JAMA	0.565**	0.782**	1.000	0.196
HON	0.208	0.103	0.196	1.000

**Correlation is significant at the 0.01 level (2-tailed). Websites ranking.

## Discussion

This study sought to evaluate the reliability and validity of the Keystone action plan criteria for assessing online medicines information. A key issue in the field of health information quality is the subjective nature of evaluations, which often rely on human judgment and various assessment tools. While many tools exist, few have been validated for online medicines information, and there is limited consensus on which criteria are most suitable for evaluating such information. The findings of this study provide valuable insights into the application of the Keystone criteria, its comparison to other widely used tools, and it’s potential to address gaps in current assessment practices (Table 5).

**Table 5:** The ranking of website according to each of the three tools.

No	Websites’ URL home addresses	Websites’ classification	Keystone (1–25)	Keystone (1–5)	DISCERN (1–25)	DISCERN (1–5)	JAMA (1–4)
W1	http://www.drugs.com	Non for profit	23	5	20	4	4
W2	http://painconcern.org.uk	Charity	1	1	1	1	1
W3	https://www.uniprix.com	Commercial	20	4	13	3	3
W4	https://www.nlm.nih.gov	Health organizations	21	5	19	4	3
W5	http://www.netdoctor.co.uk	Commercial	23	5	12	3	2
W6	http://reference.medscape.com	Not for profit	22	5	23	5	4
W7	http://www.uofmhealth.org	Academic	24	5	21	5	4
W8	http://www.news-medical.net	Commercial	18	4	11	3	3
W9	http://www.pdr.net	Commercial	2	1	8	2	2
W10	http://www.cigna.com	Health organizations	21	5	24	5	4
W11	http://www.theathlete.org	Commercial	3	1	9	2	2
W12	http://depression.emedtv.com	Commercial	11	3	14	3	2
W13	http://drugs.webmd.boots.com	Commercial	25	5	25	5	4
W14	http://www.healthyplace.com	Commercial	14	3	16	4	3
W15	http://www.mayoclinic.org	Not for profit	13	3	24	5	4
W16	https://dailymed.nlm.nih.gov	Health organizations	7	2	10	2	2
W17	http://www.wikidoc.org	Academic	10	2	21	5	4
W18	http://www.medicines.org.uk	Not for profit	6	2	5	1	1
W19	http://www.rxlist.com	Commercial	14	3	22	5	4
W20	http://www.medic-inform.com	Commercial	15	3	22	5	2
W21	http://www.home.intekom.com	Commercial	4	1	4	1	1
W22	http://www.prescriptiondrugs.com	Commercial	3	1	3	1	1
W23	http://www.anti-aging-meds.com	Commercial	17	4	21	5	4
W24	http://www.syrianclinic.com	Not for profit	6	2	6	2	2
W25	http://www.peacehealth.org	Commercial	19	4	18	4	3
W26	http://www.netdoctor.co.uk	Commercial	9	2	15	3	2
W27	http://dailymed.nlm.nih.gov	Health organizations	8	2	7	2	1
W28	http://www.news-medical.net	Commercial	16	4	17	4	3
W29	http://psychcentral.com	Charity	12	3	20	4	3
W30	http://www.druginformation.com	Commercial	5	1	2	1	1

The results of the study demonstrate that the Keystone action plan criteria exhibit strong reliability and internal consistency, outperforming other generic tools such as DISCERN, HONcode, and JAMA in assessing online medicines information (64). The Keystone criteria’s ability to consistently produce reliable results across multiple assessors, coupled with its high internal consistency, suggests that it is a robust tool for evaluating medicines-related websites. These findings reinforce the importance of considering internal consistency when choosing evaluation tools for online health information, as tools with low reliability, such as HONcode and JAMA, may not adequately capture the quality of medicines information on the web (41, 65).

Moreover, the study’s examination of inter-rater reliability using the Inter-Class Correlation (ICC) and Kendall’s rank-order correlation further supports the credibility of the Keystone criteria. The positive correlations between Keystone and other tools like DISCERN and JAMA indicate that while there are differences in how these tools measure information quality, they share common elements that ensure a level of agreement among assessors. This highlights the potential for Keystone to be used in conjunction with other instruments, expanding its applicability across various types of health information evaluation (41).

A key contribution of this study is its demonstration of the validity of the Keystone action plan criteria in assessing medicines information online. While previous research has focused on individual tools or compared generic criteria, this study is one of the first to specifically measure the validity of Keystone in the context of evaluating online medicines information. The comparison between the Keystone action plan criteria and established tools like DISCERN and JAMA has shown that the former not only aligns well with these tools but also addresses the unique challenges posed by medicines-related content, including its currency and specificity (18). This finding suggests that the Keystone criteria are not only valid for assessing general health information but are particularly effective for domain-specific evaluations.

The dynamic nature of online information presents another challenge for evaluation tools. Unlike traditional print-based health resources, web content is frequently updated, which can affect the relevance and trustworthiness of health information over time. While this study provides a snapshot of the reliability and validity of the Keystone action plan criteria, it also underscores the need for ongoing evaluations. A follow-up assessment could help to confirm the long-term effectiveness of these tools in a rapidly changing digital environment. Moreover, the study highlights the importance of developing tools that can account for updates in web content, suggesting that future research could explore automated assessment systems or artificial intelligence-based tools to mitigate the issue of time-sensitive evaluations (52).

Another noteworthy limitation of this study is its exclusive focus on English-language websites. The findings may not be directly applicable to non-English-speaking populations, as language and cultural differences can influence the structure and presentation of health information. For instance, Arabic-language health information websites may require distinct quality and reliability criteria due to linguistic and cultural factors. This limitation suggests a need for future multilingual studies to evaluate the applicability of the Keystone criteria across different languages and cultural contexts, ensuring that global health information is assessed with tools that are both reliable and relevant to diverse populations (63). Moreover, this study has a limitation in that the health literacy level of the raters was not formally evaluated. The selection of raters was based on availability, and they underwent 2 weeks of training. However, the absence of a formal health literacy assessment may restrict our comprehension of how differing levels of health literacy could affect the evaluation process. The training aimed to equip raters with the essential skills for evaluating online medical content. However, future research could enhance understanding by formally assessing health literacy and its impact on the evaluation of health information.

Despite these limitations, this study makes a significant contribution to the literature by addressing gaps in the current literature on the reliability and validity of online medicines information assessment tools. By demonstrating that the Keystone action plan criteria are both reliable and valid for evaluating online medicines information, it provides evidence that this tool can be a valuable resource for future assessments. Additionally, the study reinforces the need for specialized, domain-specific tools to evaluate medicines information online, as generic instruments may fail to address the unique characteristics of such content (52, 64).

Overall, the findings of this study suggest that the Keystone action plan criteria offer a promising approach to evaluating the quality of online medicines information. Future research should continue to explore its applicability in various contexts, considering factors such as linguistic diversity and the dynamic nature of online content. With further validation, these criteria could be instrumental in improving the quality and reliability of health information available to consumers online.

## Conclusion

Keystone action plan criteria have been designed initially to assess written information on medicines for consumers with a future expectation by its authors to be applied to online sources of information. Based on the tests performed with these criteria in this study, it can be concluded that the Keystone action plan criteria can be used to reliably assess websites information on Amitriptyline and possibly for other medicines. The criteria can be used as a domain specific tool for assessing medicines information in websites with satisfied internal consistency and reliability results that indicates the construct validity.

## Data Availability

The raw data supporting the conclusions of this article will be made available by the authors, without undue reservation.

## References

[1] Hämeen-Anttila K Pietilä K Pylkkänen L Pohjanoksa-Mäntylä M. Internet as a source of medicines information (MI) among frequent internet users. Res Soc Adm Pharm. (2018) 14:758–64. doi: 10.1016/j.sapharm.2017.09.007, PMID: 29066142

[2] Prusti M Lehtineva S Pohjanoksa-Mäntylä M Bell JS. The quality of online antidepressant drug information: an evaluation of English and Finnish language web sites. Res Soc Adm Pharm. (2012) 8:263–8. doi: 10.1016/j.sapharm.2011.03.003, PMID: 21712148

[3] Morgan M Montagne M. Drugs on the internet, part II: antidepressant medication web sites. Subst Use Misuse. (2011) 46:1628–41. doi: 10.3109/10826084.2011.588878, PMID: 21895517

[4] Nasser S Mullan J Bajorek B. Assessing the quality, suitability and readability of internet-based health information about warfarin for patients. Australas Med J. (2012) 5:194–203. doi: 10.4066/AMJ.2012.86 22952566 PMC3433734

[5] Raban MZ Tariq A Richardson L Byrne M Robinson M Li L. Evaluation of web-based consumer medication information: content and usability of 4 Australian websites. Interact J Med Res. (2016) 5:e21. doi: 10.2196/ijmr.5651, PMID: 27443680 PMC4974450

[6] Soroya SH Farooq A Mahmood K Isoaho J Zara S-e. From information seeking to information avoidance: understanding the health information behavior during a global health crisis. Inf Process Manag. (2021) 58:102440. doi: 10.1016/j.ipm.2020.102440, PMID: 33281273 PMC7700063

[7] Eysenbach G. Infodemiology: the epidemiology of (mis)information. Am J Med. (2002) 113:763–5. doi: 10.1016/S0002-9343(02)01473-0, PMID: 12517369

[8] Mueller J Jay C Harper S Davies A Vega J Todd C. Web use for symptom appraisal of physical health conditions: a systematic review. J Med Internet Res. (2017) 19:e202. doi: 10.2196/jmir.6755, PMID: 28611017 PMC5487739

[9] Charnock D Shepperd S Needham G Gann R. DISCERN: an instrument for judging the quality of written consumer health information on treatment choices. J Epidemiol Community Health. (1999) 53:105–11. doi: 10.1136/jech.53.2.105, PMID: 10396471 PMC1756830

[10] Risk A Dzenowagis J. Review of internet health information quality initiatives. J Med Internet Res. (2001) 3:E28. doi: 10.2196/jmir.3.4.e28, PMID: 11772543 PMC1761913

[11] Wilson P. How to find the good and avoid the bad or ugly: a short guide to tools for rating quality of health information on the internet. BMJ. (2002) 324:598–602. doi: 10.1136/bmj.324.7337.598, PMID: 11884329 PMC1122517

[12] Beaunoyer E Arsenault M Lomanowska AM Guitton MJ. Understanding online health information: evaluation, tools, and strategies. Patient Educ Couns. (2017) 100:183–9. doi: 10.1016/j.pec.2016.08.028 27595436

[13] Robillard JM Jun JH Lai JA Feng TL. The QUEST for quality online health information: validation of a short quantitative tool. BMC Med Inform Decis Mak. (2018) 18:87. doi: 10.1186/s12911-018-0668-9, PMID: 30340488 PMC6194721

[14] Jadad AR Gagliardi A. Rating health information on the internet: navigating to knowledge or to babel? JAMA. (1998) 279:611–4. doi: 10.1001/jama.279.8.611 9486757

[15] Gagliardi A Jadad AR. Examination of instruments used to rate quality of health information on the internet: chronicle of a voyage with an unclear destination. BMJ. (2002) 324:569–73. doi: 10.1136/bmj.324.7337.569, PMID: 11884320 PMC78993

[16] Bernstam EV Shelton DM Walji M Meric-Bernstam F. Instruments to assess the quality of health information on the world wide web: what can our patients actually use? Int J Med Inform. (2005) 74:13–9. doi: 10.1016/j.ijmedinf.2004.10.001, PMID: 15626632

[17] Zhang Y Sun Y Xie B. Quality of health information for consumers on the web: a systematic review of indicators, criteria, tools, and evaluation results. J Assoc Inf Sci Technol. (2015) 66:2071–84. doi: 10.1002/asi.23311

[18] Kim KY Metzger A Wigle PR Choe PJ. Evaluation of online consumer medication information. Res Soc Adm Pharm. (2011) 7:202–7. doi: 10.1016/j.sapharm.2010.04.003, PMID: 21272544

[19] Lankes RD. Credibility on the internet: shifting from authority to reliability. J Doc. (2008) 64:667–86. doi: 10.1108/00220410810899709

[20] Eysenbach G Diepgen TL. Towards quality management of medical information on the internet: evaluation, labelling, and filtering of information. BMJ. (1998) 317:1496–502. doi: 10.1136/bmj.317.7171.1496, PMID: 9831581 PMC1114339

[21] Bates BR Romina S Ahmed R Hopson D. The effect of source credibility on consumers' perceptions of the quality of health information on the internet. Med Inform Internet Med. (2006) 31:45–52. doi: 10.1080/14639230600552601, PMID: 16754366

[22] Pletneva N Cruchet S Simonet MA Kajiwara M Boyer C. Results of the 10 HON survey on health and medical internet use. Stud Health Technol Inform. (2011) 169:73–7. PMID: 21893717

[23] Bohacek L Gomez M Fish JS. An evaluation of internet sites for burn scar management. J Burn Care Rehabil. (2003) 24:246–51. doi: 10.1097/01.BCR.0000075844.04297.D9, PMID: 14501424

[24] Tiller G Rea S Silla R Wood F. Burns first aid information on the internet. Burns. (2006) 32:897–901. doi: 10.1016/j.burns.2006.02.020 17005324

[25] Ansani NT Vogt M Henderson BA McKaveney TP Weber RJ Smith RB. Quality of arthritis information on the internet. Am J Health Syst Pharm. (2005) 62:1184–9. doi: 10.1093/ajhp/62.11.1184, PMID: 15984050

[26] Martin-Facklam M Kostrzewa M Martin P Haefeli WE. Quality of drug information on the world wide web and strategies to improve pages with poor information quality. An intervention study on pages about sildenafil. Br J Clin Pharmacol. (2004) 57:80–5. doi: 10.1046/j.1365-2125.2003.01954.x, PMID: 14678344 PMC1884422

[27] Akbari K Som R. Evaluating the quality of internet information for bariatric surgery. Obes Surg. (2014) 24:2003–6. doi: 10.1007/s11695-014-1403-y, PMID: 25179387

[28] Kwon JH Kye SY Park EY Oh KH Park K. What predicts the trust of online health information? Epidemiol Health. (2015) 37:e2015030. doi: 10.4178/epih/e2015030, PMID: 26212505 PMC4546988

[29] Murray E Lo B Pollack L Donelan K Catania J Lee K. The impact of health information on the internet on health care and the physician-patient relationship: national U.S. survey among 1.050 U.S. physicians. J Med Internet Res. (2003) 5:e17. doi: 10.2196/jmir.5.3.e17, PMID: 14517108 PMC1550564

[30] Luo A Qin L Yuan Y Yang Z Liu F Huang P. The effect of online health information seeking on physician-patient relationships: systematic review. J Med Internet Res. (2022) 24:e23354. doi: 10.2196/23354, PMID: 35142620 PMC8874798

[31] Holloway K Bennett T. Prescription drug misuse among university staff and students: a survey of motives, nature and extent. Drugs. (2012) 19:137–44. doi: 10.3109/09687637.2011.594114

[32] Weisbord SD Soule JB Kimmel PL. Poison on line—acute renal failure caused by oil of wormwood purchased through the internet. N Engl J Med. (1997) 337:825–7. doi: 10.1056/NEJM199709183371205, PMID: 9297113

[33] Crocco AG Villasis-Keever M Jadad AR. Analysis of cases of harm associated with use of health information on the internet. JAMA. (2002) 287:2869–71. doi: 10.1001/jama.287.21.2869 12038937

[34] Fadhel F. Misuse of prescription drugs and other psychotropic substances among university students: a pilot study. East Mediterr Health J. (2022) 28:288–95. doi: 10.26719/emhj.22.019, PMID: 35545910

[35] Raine C Webb DJ Maxwell SR. The availability of prescription-only analgesics purchased from the internet in the UK. Br J Clin Pharmacol. (2009) 67:250–4. doi: 10.1111/j.1365-2125.2008.03343.x, PMID: 19154446 PMC2670383

[36] Royds J Conroy MJ Dunne MR McCrory C Lysaght J. An investigation into the modulation of T cell phenotypes by amitriptyline and nortriptyline. Eur Neuropsychopharmacol. (2020) 31:131–44. doi: 10.1016/j.euroneuro.2019.12.106, PMID: 31882254

[37] Thour A Marwaha R. *Amitriptyline. StatPearls NCBI bookshelf*. (2023). 30725910

[38] Mononen N Järvinen R Hämeen-Anttila K Airaksinen M Bonhomme C Kleme J. A national approach to medicines information research: a systematic review. Res Soc Adm Pharm. (2018) 14:1106–24. doi: 10.1016/j.sapharm.2018.01.011, PMID: 29483046

[39] Alhlayl A. The development and evaluation of a computer-based tool for assessing web-based information on medicines. Swansea: Swansea University (2019).

[40] Read J. Antidepressants and suicide: 7,829 inquests in England and Wales, 2003–2020. Ethical Hum Psychol Psychiatry. (2023) 25:8–28. doi: 10.1891/EHPP-2022-0015

[41] Heald AH Stedman M Davies M Livingston M Taylor D Gadsby R. Antidepressant prescribing in England: patterns and costs. Prim Care Companion CNS Disord. (2020) 22:26631. doi: 10.4088/PCC.19m02552, PMID: 32302071

[42] Griffiths KM Tang TT Hawking D Christensen H. Automated assessment of the quality of depression websites. J Med Internet Res. (2005) 7:e59. doi: 10.2196/jmir.7.5.e59, PMID: 16403723 PMC1550680

[43] Hertzog MA. Considerations in determining sample size for pilot studies. Res Nurs Health. (2008) 31:180–91. doi: 10.1002/nur.20247 18183564

[44] Wang L Wang J Wang M Li Y Liang Y Xu D. Using internet search engines to obtain medical information: a comparative study. J Med Internet Res. (2012) 14:e74. doi: 10.2196/jmir.1943, PMID: 22672889 PMC3799567

[45] Al-Ubaydli M. Using search engines to find online medical information. PLoS Med. (2005) 2:e228. doi: 10.1371/journal.pmed.0020228, PMID: 16022563 PMC1181536

[46] Arif N Ghezzi P. Quality of online information on breast cancer treatment options. Breast. (2018) 37:6–12. doi: 10.1016/j.breast.2017.10.004, PMID: 29040893

[47] Arora VS McKee M Stuckler D. Google trends: opportunities and limitations in health and health policy research. Health Policy. (2019) 123:338–41. doi: 10.1016/j.healthpol.2019.01.001, PMID: 30660346

[48] Huynh Dagher S Lamé G Hubiche T Ezzedine K Duong TA. The influence of media coverage and governmental policies on Google queries related to COVID-19 cutaneous symptoms: Infodemiology study. JMIR Public Health Surveill. (2021) 7:e25651. doi: 10.2196/25651, PMID: 33513563 PMC7909455

[49] Fahy E Hardikar R Fox A Mackay S. Quality of patient health information on the internet: reviewing a complex and evolving landscape. Australas Med J. (2014) 7:24–8. doi: 10.4066/AMJ.2014.1900, PMID: 24567763 PMC3920473

[50] Al-Jefri MM Evans R Ghezzi P Uchyigit G. *Using machine learning for automatic identification of evidence-based health information on the web*. Proceedings of the 2017 international conference on digital health (2017).

[51] Charnock D Shepperd S. Learning to DISCERN online: applying an appraisal tool to health websites in a workshop setting. Health Educ Res. (2004) 19:440–6. doi: 10.1093/her/cyg046, PMID: 15155597

[52] Charnock D. The DISCERN handbook: Quality criteria for consumer health information on treatment choices. Oxon: Radcliffe Medical Press (1998).

[53] Eysenbach G Thomson M. The FA4CT algorithm: a new model and tool for consumers to assess and filter health information on the internet. Stud Health Technol Inform. (2007) 129:142–6. 17911695

[54] Charnock D. The DISCERN handbook. Quality criteria for consumer health information on treatment choices Radcliffe. Oxford: University of Oxford and The British Library, pp. 7–51. (1998).

[55] Charnock D Shepperd S. *The DISCERN instrument: Radcliffe online*. (2012). Available at: http://www.discern.org.uk/discern_instrument.php.

[56] HON. *Principles—quality and trustworthy health information*. (2023). Available at: https://www.hon.ch/en/certification.html.

[57] Boyer C Gaudinat A Hanbury A Appel RD Ball MJ Carpentier M. Accessing reliable health information on the web: A review of the HON approach. Stud Health Technol Inform. (2017):1004–8. PMID: 29295252

[58] Boyer C Frossard C Gaudinat A Hanbury A Falquetd G. How to sort trustworthy health online information? Improvements of the automated detection of HONcode criteria. Proc Comput Sci. (2017) 121:940–9. doi: 10.1016/j.procs.2017.11.122

[59] Ahmed W. Evaluation of web-sites that contain information relating to the norovirus infection and the evaluation of generic and specific instruments deployed to evaluate web-sites. Sheffield: University of Sheffield (2013).

[60] Rothrock SG Rothrock AN Swetland SB Pagane M Isaak SA Romney J. Quality, trustworthiness, readability, and accuracy of medical information regarding common pediatric emergency medicine-related complaints on the web. J Emerg Med. (2019) 57:469–77. doi: 10.1016/j.jemermed.2019.06.043, PMID: 31561928

[61] Santos J. Cronbach’s alpha: a tool for assessing the reliability of scales. J Ext. (1999) 37:1–5.

[62] Bonett DG Wright TA. Cronbach's alpha reliability: interval estimation, hypothesis testing, and sample size planning. J Organ Behav. (2015) 36:3–15. doi: 10.1002/job.1960

[63] Shrout PE Fleiss JL. Intraclass correlations: uses in assessing rater reliability. Psychol Bull. (1979) 86:420–8. doi: 10.1037/0033-2909.86.2.420 18839484

[64] Khamis H. Measures of association: how to choose? J Diagn Med Sonogr. (2008) 24:155–62. doi: 10.1177/8756479308317006

[65] Pereira CDS Cruz JN Ferreira MKM Baia-da-Silva DC Fontes-Junior EA Lima RR. Global research trends and hotspots analysis of the scientific production of amitriptyline: a bibliometric approach. Pharmaceuticals. (2023) 16:47. doi: 10.3390/ph16071047, PMID: 37513958 PMC10386017

